# The Consumer’s Reservation Price as an Adaptive Aspiration Level

**DOI:** 10.3390/bs16030421

**Published:** 2026-03-13

**Authors:** Sebastian van Baal

**Affiliations:** 1 Faculty of Economics, CBS International Business School, Bahnstrasse 6-8, 50996 Cologne, Germany; s.vanbaal@cbs.de; 2 German Economic Institute, Konrad-Adenauer-Ufer 21, 50668 Cologne, Germany

**Keywords:** behavioral economics, bounded rationality, satisficing, consumer behavior, search theory, reservation price, aspiration level, D91, D83, C91

## Abstract

Reservation prices determine which goods consumers are willing to buy and, therefore, shape demand curves in markets. Neoclassical economics postulates that reservation prices optimally reflect the marginal utility provided by a good given all other possible uses of the consumer’s budget, as well as a rational response to the information environment. In contrast, behavioral economics suggests that reservation prices are influenced by extraneous factors and are, thus, less stable and more difficult to predict. In this article, I propose a behavioral model of how the reservation price changes during sequential price searches. The model assumes bounded rationality, is rooted in the psychological theory of aspiration levels, and posits that the reservation price adjusts towards the lowest price known. A corollary is that when higher prices are charged in a market, consumers become willing to pay more in the short term. Results from an online laboratory experiment with more than 400 participants from the general population suggest that the model performs well in explaining the dynamics of the reservation price during a search spell. While the results imply that reservation prices are malleable, competition can protect consumers from sellers exploiting their adaptiveness.

## 1. Introduction

Consider a consumer who wants to buy a specific good, e.g., a refrigerator. She is willing to pay 600 euros, her reservation price. In a store, she finds the refrigerator for 800 euros, and reluctantly becomes willing to pay 700 euros. On the manufacturer’s website, she finds a price of 1000 euros, revising her reservation price again to 750 euros. After getting more price information, she ends up buying the refrigerator for 720 euros, 20 percent more than what she initially wanted to spend.

Such casually observed adaptations of reservation prices are the subject of this article. This research is motivated by the contrast between the neoclassical microeconomic theory of demand, which posits that consumers make rational decisions and therefore determine reservation prices optimally, and behavioral economics, which is based on bounded rationality and therefore does not assume optimization. Before specifying the article’s contribution, I outline this general background.

In the microeconomic theory of demand, the reservation price is a central construct (e.g., Varian, 1992). In essence, a consumer buys a discrete good only if its price is not higher than the consumer’s reservation price for the good. In terms of preference ordering, the consumer prefers keeping any amount of money higher than the reservation price to obtaining the good; the consumer prefers the good to any amount of money less than the reservation price. Under the cardinal interpretation of utility, the reservation price reflects the monetary valuation of the marginal utility the consumer associates with the good given all other possible uses of the budget. As long as consumers have reservation prices, sellers face downward-sloping or horizontal demand curves, and expected sales are therefore a non-increasing function of price, which is a necessary condition for markets to work in the public interest by driving prices down towards marginal cost.

Largely based on research in behavioral economics, marketing science, and cognitive psychology, doubts have been raised as to whether consumers actually have stable preferences and, by extension, reservation prices (e.g., Ariely et al., 2003; Bettman et al., 1998; Hoeffler & Ariely, 1999; Payne et al., 1992; Tversky & Simonson, 1993; see also Gilboa & Schmeidler, 2001). A main alternative theory is that consumers construct their preferences based on uninformative cues, where “uninformative” refers to the utility provided by the good. Given the myriad of potential uninformative cues or “supposedly irrelevant factors” (Thaler, 2015), modeling the construction of consumer preferences becomes both more challenging and more important. If reservation prices are not stable, our understanding of markets can be enhanced by developing models of underlying behavioral dynamics.

In this article, I propose and experimentally test such a model of the dynamics of the reservation price during a consumer’s search for prices. The model is inspired by Simon’s behavioral model of rational choice (Simon, 1955) and their satisficing heuristic (Simon, 1956), which initiated the research on bounded rationality and are rooted in the psychological theory of aspiration levels, as outlined by Lewin et al. (1944). Since an aspiration level is an operational goal for an action, i.e., the performance an individual strives to attain, it is akin to the reservation price (see, e.g., Artinger et al., 2022), i.e., the price a consumer strives to find for a good when searching for offers. Hence, the theory of aspiration levels is the foundation of the model presented here, and the research question is whether the reservation price changes during price search in accordance with this theory, which posits a relatively simple adjustment process. In contrast, neoclassical search models assume rationality and therefore optimization, which implies extensive information processing capabilities on the part of consumers (e.g., Hey, 1981; Rothschild, 1974).

To preview the findings, the essence of the behavioral dynamics suggested by applying the theory of aspiration levels to the reservation price is fairly intuitive. When consumers sequentially search for prices, i.e., canvass sellers one after the other to identify an acceptable price for a good they exogenously decided to purchase, the prices they observe during their search are potential uninformative cues (see also Isoni et al., 2016; Mazar et al., 2014). The main hypothesis of the model is that consumers’ reservation prices adjust during search towards the lowest price known: if the minimum price identified thus far is higher (lower) than the reservation price, the reservation price increases (decreases). Thus, the reservation price is adaptive to the prices observed during their search, which parallels the adaptive nature of the generic concept of aspiration levels (Lewin et al., 1944). In addition, the model incorporates ancillary hypotheses derived from the theory of aspiration levels, in particular, an asymmetric reaction to higher vs. lower minimum prices and a tendency to reduce the reservation price during their search. Results from an online laboratory experiment with more than 400 participants from the general population suggest that the model performs well in explaining the dynamics of the reservation price.

This article proceeds as follows. The pertinent literature with respect to sequential price search, unstable preferences, and other extant research related to the dynamics of the reservation price is reviewed in Section 2. In Section 3, after a brief overview of the theory of aspiration levels, the main model of the present research is developed. The experimental method used to test the model is described in Section 4, followed by the results in Section 5. Implications and limitations are discussed in Section 6, and Section 7 concludes.

## 2. Literature Review and Research Gap

### 2.1. The Reservation Price in Neoclassical Search Theory

In the seminal search model by Stigler (1961), consumers decide before search begins how many sellers to visit. Such fixed-sample-size models (Manning & Morgan, 1982; Rothschild, 1974) are based on order statistics that are processed by consumers ex ante, which means that the prices identified during search do not affect search behavior. In other words, search is not sequential, as consumers do not decide in each search step whether to continue or stop their search. Therefore, there is no reservation price (but only a fixed sample size) that guides search in such models. In some situations, this may be an appropriate model (e.g., Manning & Morgan, 1982), but when a consumer searches for prices, a fixed-sample-size rule typically is neither rational nor seems compatible with common sense (Rothschild, 1974). In sequential search models, which seem more fitting, search is stopped once a price is identified that is not higher than a threshold, and this threshold is the reservation price (see generally Weitzman, 1979). Under the neoclassical assumption of rationality, consumers determine the optimal reservation price $RP^{*}$ based on the cumulative price distribution F(p) and marginal search cost *C* according to the following equation (McCall, 1970; Rothschild, 1974):

(1) $$\int_{0}^{RP^{*}}F\left(p\right)\;dp=C$$

In such models, consumers determine $RP^{*}$ before search begins, which means that the reservation price does not change during search. This requires consumers knowing F(p) ex ante, which is, in many cases, an unrealistic assumption. (An alternative assumption is that consumers behave as if they know F(p)—but this point of view invokes the usual criticism of the as-if assumption; e.g., Conlisk, 1996). Search models with an unknown price distribution are more heterogeneous: there is no unifying approach for this setting. A representative model, based on Bayesian statistics, is the one by Rothschild (1974; for generalizations, see, e.g., Bikhchandani & Sharma, 1996). An essential element of their model is a searcher’s optimal strategy when the prices identified during search convey information about the parameters of the price distribution. Not in all, but in many cases, rational searchers determine a reservation price according to the following equation:

(2) $$RP_{i}^{*}=V\left[h_{i}\left(\mu ,\rho \right)\right]+C$$

$RP_{i}^{*}$ is the optimal reservation price when price *i* is observed, and $V[h_{i}\left(\mu ,\rho \right)]$ is—in simplified terms—the expected price when carrying out one more search step (including the value of the option to carry out even more steps) if the searcher’s knowledge of prices is summarized in μ (the content of the information) and ρ (the precision) and the searcher updates their beliefs according to a rule $h_{i}$. Hence, in this model, the reservation price is dynamic in the sense that it may respond to the prices observed during search (see also, for example, Axell, 1974; Karni & Schwartz, 1977; Kohn & Shavell, 1974). Although this feature of the model is similar to the main hypothesis of the present research, its foundation and implications are different. Rothschild (1974) shows the intricate mathematical operations a consumer would need to implicitly perform to behave as if in accordance with their model (see also Hey, 1981), whereas the model presented in this study does not suggest extensive information processing capabilities or attempts to learn about the price distribution in a statistical sense. While the fragmented literature on optimal search with an unknown price distribution includes other attempts to model computationally less demanding strategies (e.g., Bikhchandani & Sharma, 1996; Chou & Talmain, 1993), they generally rely on concepts from mathematical statistics rather than emphasizing behavioral foundations. (For models that do not assume optimizing behavior, see Section 2.3.2.)

In general, sequential search models differ as to whether they allow searchers to exercise a recall, i.e., to go back to accept a price which they previously rejected in the search spell. In neoclassical models with a known price distribution, recalls cannot occur because the optimal reservation price is static (e.g., Rosenfield & Shapiro, 1981; Telser, 1973). Models with an unknown price distribution are more varied and complex in this respect. For instance, Rothschild (1974) assumes that recalls are not possible, while Kohn and Shavell (1974) focus on the opposite and Karni and Schwartz (1977) analyze the intermediate case. Another variation in sequential search models concerns the number of search steps that can be carried out: in models with an infinite search horizon, there is no upper limit (e.g., Kohn & Shavell, 1974), while in models with a finite search horizon, there is (e.g., Rothschild, 1974).

With respect to consumer price search, it seems realistic to assume that searchers typically do not know the price distribution, at least not in a statistical sense, and especially for first-time buyers; that the search horizon is finite, since there is usually an upper limit to the number of stores, for example, that can be visited in any given time frame; and that recalls are possible, provided that prices stay constant during a search spell (Axell, 1974; Hey, 1981; Karni & Schwartz, 1977; Kohn & Shavell, 1974; Marcu & Noussair, 2024; Rothschild, 1974; Schunk & Winter, 2009; Stigler, 1961; Telser, 1973). The present research is based on these assumptions. In addition to their presumed realism, they are motivated by Hey’s observation that the neoclassical “model of optimal search is not a particularly good description of absolute behaviour, and that behaviour worsens when information lessens and when the facility of recall is introduced” (Hey, 1991, p. 110; see also Hey, 1987, 1993; Casner, 2021). Hence, this setting in particular calls for an alternative model that does not assume optimizing behavior.

### 2.2. Unstable Preferences and the Reservation Price

The debate as to whether consumers or, more generally, decision makers can be assumed to behave rationally has a long tradition, and an instance of this debate is whether preferences are stable in the sense that they do or do not respond to normatively irrelevant factors (e.g., Simon, 1955, 1956, 1979; Antonides, 1996; Bettman et al., 1998; Gigerenzer, 2018; Payne et al., 1992; Tversky & Simonson, 1993). While both points of view have scientific merit and while one may invoke several arguments for the assumption of rationality (see the discussion by Conlisk, 1996), there is evidence that consumer preferences are often unstable. For example, consumers are influenced by extraneous price information: they are willing to pay more, i.e., their reservation price rises, for a compact disc when the price of an unrelated sweatshirt offered by an adjacent vendor is higher (Nunes & Boatwright, 2004). A well-known experimental observation by Ariely et al. (2003; see also, for example, Ariely et al., 2006) is that reservation prices for common goods such as computer peripherals or wine and uncommon goods such as the avoidance of an annoying sound can be influenced by one’s social security number if the latter is made salient as an arbitrary anchor (Tversky & Kahneman, 1974). A third example is the seminal observation by Thaler (1985, 1999) that a consumer’s reservation price for a bottle of beer is higher when the bottle is obtained from a fancy hotel rather than from a run-down store even if the ultimate consumption experience is the same, which can be attributed to different mental accounts and reference prices (see Section 2.3.1) for the different sellers. In light of such findings, it is not impossible but at least difficult to maintain the opinion that reservation prices reflect a rational allocation of consumers’ resources (and an optimal response to the information environment), as presumed in neoclassical search theory. The behavioral dynamics of the reservation price are likely to be more subtle psychologically, but not necessarily computationally.

### 2.3. Additional Perspectives on the Dynamics of the Reservation Price

#### 2.3.1. Reference Prices

A construct that is not in general emphasized in economic theory but scrutinized in marketing research is a consumer’s reference price (e.g., Winer, 1988). While there are several ways to conceptualize this multidimensional construct, one of which is the reservation price, a standard interpretation is that it represents the price a consumer considers “normal” or “fair” for a certain good; a regular foundation for this interpretation is adaptation-level theory (Helson, 1964). Since an adaptation level is based on experiences, the reference price can be dynamic in that it adapts to the prices observed over time. A common model of this process is based on adaptive expectations (Winer, 1988; generally Arrow & Nerlove, 1958) and can be expressed as Equation (3) ($REF_{t}$ is the reference price and $P_{t}$ is the price observed at time *t*; *b* is a weighting parameter).

(3) $$REF_{t}=b\;REF_{t-1}+\left(1-b\right)P_{t-1}$$

In principle, the adaptation of the reference price parallels the main hypothesis of the present research with respect to the reservation price. However, there are two differences: First, although the reference price as the subjective “normal” or “fair” price influences consumers’ *judgments* of prices, it does not determine the *decision* whether to make a purchase, while the reservation price does (see also Section 3.1.1; Jacobson & Obermiller, 1989; Lichtenstein et al., 1988). A positive difference between a consumer’s reference price and an observed price may lead the consumer to evaluate the price as “justified,” but does not necessarily lead to a purchase (e.g., Wertenbroch & Skiera, 2002, show that a consumer’s reservation price is not the same as the price normally paid for a good). Second, studies of reference price formation suggest that it is a relatively long-term phenomenon, i.e., the adaptation occurs *between* purchases (see Jacobson & Obermiller, 1989, and the sources cited therein). The present research studies the short-term adaptation *within* a purchase (i.e., search spell).

#### 2.3.2. Non-Optimizing Search Behavior

Given the vastness of research related to information acquisition by consumers, this literature review is necessarily selective. One strain that is connected to neoclassical search theory and relevant for the present study includes the works by Hey (1981): based on a criticism of the information processing capabilities assumed in models of optimizing behavior, he proposes a set of rules of thumb that searchers may actually follow (Hey, 1981, 1982, 1987, 1991). One of these rules invokes a (possibly changing) non-optimal reservation price, but Hey (1982) does not aim to explain how it is determined. In connection to these works, Butler and Loomes (1997) develop an “aspiration-satisficing” model of search behavior, which is similar in spirit to the objective of the present research. They formulate several variants of their model; the one that puts the least cognitive demands on the searcher is analogous to Equation (4) ($RP_{t}$ is the reservation price at time *t*; there is an apparent similarity to Equation (3)).

(4) $$RP_{t}=b\;RP_{t-1}+\left(1-b\right)P_{t}$$

Butler and Loomes (1997) find experimental support for their model, but their evidence is—as they remark—not conclusive. In addition, their approach is rather exploratory in that its theoretical foundations are no more than sketched. Thus, while the findings by Butler and Loomes (1997) suggest that it is worthwhile to consider a model based on the theory of aspiration levels, a more detailed and theoretically founded analysis is called for.

## 3. The Consumer’s Reservation Price as an Adaptive Aspiration Level

### 3.1. The Theory of Aspiration Levels and Its Application to the Reservation Price

#### 3.1.1. The Aspiration Level as an Independent Variable

According to the theory of aspiration levels, an individual’s evaluation of a performance in an action not only depends on the performance itself, but also on the level of aspiration with respect to the performance (Dembo, 1931; Hoppe, 1930; Lewin et al., 1944; “performance” and “action” are to be understood in a broad sense). Thus, the aspiration level serves as a reference point: Subjective evaluations are formed relative to the aspiration level. If the aspiration level is achieved, the performance is deemed a success; if it is not achieved, it is a failure (Lewin et al., 1944). However, aspiration levels are not the same as adaptation levels (Helson, 1964, especially pp. 395–400), which are often the theoretical foundation for reference points: The aspiration level reflects the level of performance an individual aspires to attain, and not necessarily the “normal” or a similarly conceptualized level of performance. Thus, the aspiration level is neither an expectation about the state of nature nor only an anchor that influences judgment, but also an operational goal for an action or decision (Lant, 1992; Lewin et al., 1944; Siegel, 1957; Starbuck, 1963). It is, therefore, likely to have a more direct impact on behavior.

Relating the psychological theory of aspiration levels to theory building in economics, Simon (1955, p. 105; see also Artinger et al., 2022; Caplin et al., 2011; Simon, 1979) notes that aspiration levels are particularly fitting to model situations in which individuals “receive a sequence of offers.” Thus, when a consumer sequentially searches for prices (the “action”), their reservation price can be interpreted as an aspiration level, with a downward orientation^1^: the consumer’s assumed goal is to find a minimum price (the “performance”) that is not higher than their reservation price. When this goal is achieved (“success”), the consumer becomes willing to end the search by making a purchase. As long as all identified prices are higher than the reservation price (“failure”), the consumer does not make a purchase and continues the search. In other words, the consumer satisfices (Simon, 1956) by attempting to find a price that is “good enough” relative to the reservation price rather than attempting to find the minimum price in the market. This decision-making strategy is also pursued in neoclassical models of sequential search, which, however, assume optimizing behavior (see Section 2.1 and Conlisk, 2003 for a connection between optimizing and satisficing search behavior). In fact, the appendix to Simon’s (1955) paper shows a model that is similar to neoclassical models with a known price distribution—and while Simon (1955) includes this model to show how the optimal reservation price would be determined rationally, the point he makes is that the “procedure would…rapidly complicate the problem beyond the computational capacity of the organism” (p. 113; see also Artinger et al., 2022; Simon, 1979). Hence, the question is how the reservation price is determined under realistic bounds on rationality, i.e., what the aspiration level as a psychological construct rather than as a mathematical solution to an optimization problem depends on.

#### 3.1.2. The Aspiration Level as a Dependent Variable

Numerous factors influence the performance an individual aspires to attain in an action. For analytical purposes and to clarify the boundaries of the present study, the beginning of an action and the process that occurs during the action can be separated. When an action begins, the (often vague) initial aspiration level can be affected by the following: experiences in similar activities, e.g., with respect to the difficulty of the task; the performance the individual assumes to be possible “objectively” due to the nature of the action; external information about possible performances, e.g., gathered by social comparison; and individual characteristics and cultural norms, e.g., regarding competitiveness (Lewin et al., 1944). While these factors can be adapted for hypotheses concerning the reservation price at the beginning of a search spell (e.g., the initial reservation price may rise in marginal search cost, as these reflect the difficulty of finding a low price; see Simon, 1955), the present study focuses on the dynamics of the reservation price *during* search. The initial reservation price enters the model as an exogenous variable.

A general hypothesis regarding the dynamics of the aspiration level during an action is that after a success, the aspiration level increases, and after a failure, it decreases (Lewin et al., 1944). Thus, the aspiration level is adaptive to actual performance. Lewin et al. (1944) semi-formalized this hypothesis in their “attainment discrepancy model” (ADM), which can be operationalized with the following equation (Lant, 1992):

(5) $$ASP_{t}=b_{0}+b_{1}ASP_{t-1}+b_{2}\left(PER_{t-1}-ASP_{t-1}\right)$$

$ASP_{t}$ is the aspiration level and $PER_{t}$ is the performance at a discrete point in time *t*; the $b_{i}$ values are theoretical model parameters. The core of the model is the difference $PER_{t-1}-ASP_{t-1}$, the “attainment discrepancy.” If $0<b_{2}\leq 1$, the aspiration level adjusts to performance (partially if the last inequality applies strictly).

Empirical studies suggest that the ADM is a valid representation of the dynamics in goal-directed behavior (e.g., Glynn et al., 1991; Lant, 1992; Mezias et al., 2002; Murphy et al., 2001). However, these studies relate to organizational behavior, not to consumer behavior. This tends to hold true for the theory of aspiration levels in general: although there are exceptions (see, e.g., Antonides, 1996), the theory is more prominent in the study of organizations (e.g., Cyert & March, 1992; March & Simon, 1993; Simon, 1997; see also Artinger et al., 2022). Whether or not the ADM is useful in explaining consumer behavior is under-researched. Therefore, I use Equation (5) as a starting point for a model of the dynamics of the consumer’s reservation price.

### 3.2. An Attainment Discrepancy Model of the Dynamics of the Consumer’s Reservation Price

Since the theory of aspiration levels and the ADM pertain to goal-directed behavior in general, of which the search for prices is a specific instance, and since consumer behavior differs from organizational behavior, Equation (5) needs to be refined. To ease the exposition, it is helpful to recast the equation in terms of the reservation price as follows:

(6) $$RP_{t}=b_{0}+b_{1}RP_{t-1}+b_{2}\left(P_{t-1}^{\min}-RP_{t-1}\right)$$

In this and subsequent equations, $RP_{t}$ is the reservation price in search step *t*; $P_{t}^{\min}:=\min\left(P_{1},P_{2},\ldots ,P_{t}\right)$ is the lowest price known, with $P_{t}$ being the price identified in search step *t*; and t=1,2,…,T indexes the search steps, with *T* referring to the end of a search spell and, thus, the total number of prices identified. Equation (6) implies that the consumer enters search step *t* (e.g., visits a store) with the intention to purchase the good only if $P_{t}^{\min}\leq RP_{t}$. In terms of the theory of aspiration levels, $P_{t}^{\min}\leq RP_{t}$ is a success and $P_{t}^{\min}>RP_{t}$ is a failure.

The first refinement to Equation (6) concerns the sequencing of events. In the ADM and its application to organizational goal setting, it is presumed that $ASP_{t}$ is determined before $PER_{t}$ occurs (Lant, 1992; Lewin et al., 1944). Hence, $PER_{t}$ cannot affect $ASP_{t}$, but only $ASP_{t+1}$. However, Lewin et al. (1944) note that an individual’s ex-post statement on $ASP_{t}$ may reflect a rationalization of $PER_{t}$, which implies that from the individual’s perspective, $PER_{t}$ may alter $ASP_{t}$ if the individual does not consider $ASP_{t}$ a commitment. When a consumer searches for prices, $RP_{t}$ reflects a goal, but not a commitment. Therefore, $P_{t}$ and, thus, $P_{t}^{\min}$ can affect $RP_{t}$: the price from the current search step may alter the reservation price for that step (see also Butler & Loomes, 1997; Jacobson & Obermiller, 1989; Karni & Schwartz, 1977; Kohn & Shavell, 1974; Rothschild, 1974). In other words, a price can affect its own acceptability, particularly if it is the lowest price observed thus far. Hence, the modification is to replace $P_{t-1}^{\min}$ with $P_{t}^{\min}$.

The second refinement models a potential asymmetry by splitting up $b_{2}$ into two parameters, $b_{2}^{+}$ and $b_{2}^{-}$, the first for upward and the second for downward adjustments of the reservation price ($P_{t}^{\min}\geq RP_{t-1}$ or, equivalently, $\max\left(0,P_{t}^{\min}-RP_{t-1}\right)$ vs. $P_{t}^{\min}\leq RP_{t-1}$ or $\min\left(0,P_{t}^{\min}-RP_{t-1}\right)$). This modification reflects the postulate by Lewin et al. (1944) that the valence (i.e., utility; Siegel, 1957; Starbuck, 1963) of failure is not always the inverse of the valence of success. Because of this potential asymmetry, any application of the theory of aspiration levels would benefit in terms of explained variation from modeling positive and negative adjustments separately (Mezias, 1988; Murphy et al., 2001; Simon, 1955). Such a separation seems particularly warranted in a model of the dynamics of the reservation price: observing that the reservation price adjusts downward when low prices are observed is not as relevant a result (e.g., for consumer policy) as observing that it adjusts upward when high prices are observed because the latter implies that consumers become willing to pay more when higher prices are charged in the market.

These refinements lead to Equation (7), which is the main model of the present research.

(7) $$\begin{array}{cc} RP_{t}=b_{0} & +b_{1}RP_{t-1}\\ & +b_{2}^{+}\left[\max\left(0,P_{t}^{\min}-RP_{t-1}\right)\right]\\ & +b_{2}^{-}\left[\min\left(0,P_{t}^{\min}-RP_{t-1}\right)\right] \end{array}$$

If the dynamics of the reservation price can be explained by the theory of aspiration levels, the following parameter hypotheses should hold:

- $b_{0}<0$: Lewin et al. (1944) note that individuals tend to set a high aspiration level to keep it above actual performance, and to increase it during an action (see also Lant, 1992; Murphy et al., 2001). This observation translates to consumers having a tendency to reduce their reservation price during search, conditional on the prices observed.
- $0<b_{1}\leq 1$: This “inertia” term models the extent to which the previous reservation price anchors the current reservation price. If there is inertia, $b_{1}>0$ (Lant, 1992). A rigid interpretation of the ADM suggests the more specific hypothesis $b_{1}=1$: if the attainment discrepancy—i.e., the difference between the lowest price known and the previous reservation price—explains changes in the reservation price, the latter should stay constant if the former is held constant. However, the tendency to reduce the reservation price may lead to $b_{1}<1$. (While $b_{0}<0$ reflects an absolute reduction in the reservation price from one search step to the next, $b_{1}<1$ reflects a relative reduction.)
- $0<b_{2}^{+}\leq 1$ and $0<b_{2}^{-}\leq 1$: The core hypothesis is that the reservation price adapts to the lowest price known, both upward and downward. Adjustments in either direction will never be perfect, which would imply a constant $b_{2}^{+}=b_{2}^{-}=1$. Upward adjustments may typically be partial ($0<b_{2}^{+}<1$), as consumers will not always raise their reservation price to the lowest price they know. Downward adjustments may also be partial ($0<b_{2}^{-}<1$) because even though consumers will likely purchase at $P_{T}^{\min}$ (Section 3.3), their reservation price can remain above the lowest price they know due to psychological stickiness, a desire to achieve greater success or to avoid future failure by keeping a buffer, and a tolerance up to just noticeable differences (see Gilboa & Schmeidler, 2001). Generally, the theory of aspiration levels implies typically gradual, not instantaneous, complete adaptation (Lewin et al., 1944; Simon, 1955).
- $b_{2}^{+}<b_{2}^{-}$: This relation operationalizes the hypothesis that downward adjustments of the reservation price are stronger than upward adjustments, reflecting the observation by Lewin et al. (1944, p. 373) that success “should make for a less tense emotional situation than failure” and that individuals have a tendency to avoid failure. This “failure aversion” is akin to loss aversion in prospect theory; see Section 6.2.2.

### 3.3. Necessary and Sufficient Conditions for Search to End with a Purchase

Although the objective of the present study is limited to explaining the dynamics of the reservation price during search, the model would be incomplete without a specification of the conditions leading a consumer to end their search with a purchase. I propose two conditions: Prior research suggests that consumers often consult at least a few sellers to learn about market prices (Stigler, 1961; Telser, 1973; Hey, 1981, 1982; Casner, 2021; Kogut, 1992; Sonnemans, 1998). Hence, the first necessary condition for the consumer to end their search is that they have identified a number of prices they consider sufficient for making a decision (Stopping Condition 1). The second necessary condition is implied by the existence of a reservation price—the consumer will end their search with a purchase only if $P_{t}^{\min}\leq RP_{t}$ (Stopping Condition 2).

Stopping Condition 1 entails that there are consumers who continue to search even if an acceptable price is found, especially in early steps. Therefore, there can be several downward adaptations of the reservation price within a search spell, as search is not necessarily concluded once $P_{t}^{\min}\leq RP_{t}$ occurs. Stopping Condition 2 does not imply that the consumer purchases the good at $P_{T}$, where *T* indexes the last search step. Instead, assuming only minimum rationality, it is plausible that the consumer purchases at $P_{T}^{\min}$ (Butler & Loomes, 1997; Hey, 1981; Kohn & Shavell, 1974; Rosenfield & Shapiro, 1981; Rothschild, 1974; Stigler, 1961; Telser, 1973; Weitzman, 1979). Hence, if the reservation price has risen enough to render the best price observed so far acceptable, the consumer will end their search by exercising a recall whenever $P_{T}^{\min}<P_{T}$.

**Figure 1:**
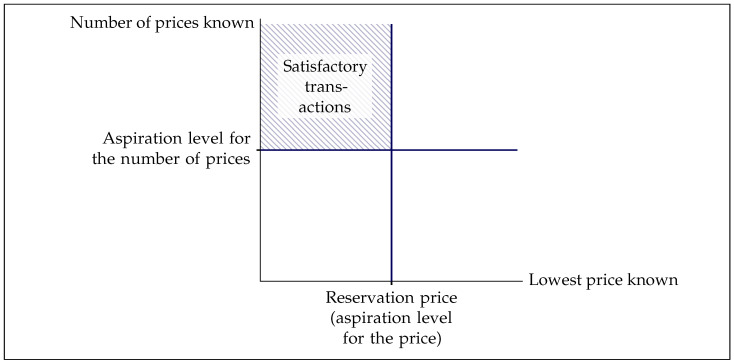
The reservation price as an independent variable. Note: This figure visualizes the two stopping conditions from Section 3.3. Adapted from Simon (1955, p. 109).

The conjunction of Stopping Conditions 1 and 2 is the sufficient condition for search to end with a purchase: if both are fulfilled in search step *t*, t≡T. In the words of Simon (1955, p. 109; see also Artinger et al., 2022; Selten, 1998), this sufficient condition means that the consumer “may be trying to implement a number of *values that do not have a common denominator*”—i.e., the searcher attempts to satisfy *two* aspiration levels, one for the price and one for the number of prices (Figure 1), both of which can be adaptive. In the present study, I do not attempt to falsify or further specify these conditions, but rather focus on the adaptiveness of the reservation price during search as part of Stopping Condition 2.

### 3.4. An Illustration of the Hypothesized Search Behavior

**Figure 2:**
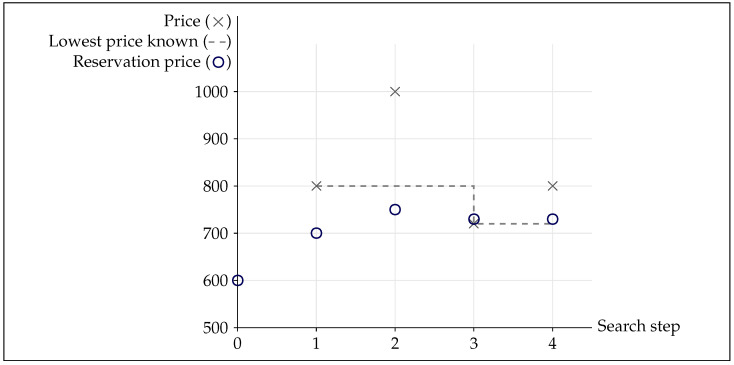
The reservation price as a dependent variable. Note: This figure visualizes the example from Section 3.4.

For a typical sequence of events and coming back to the opening vignette from Section 1, consider a consumer wanting to buy a refrigerator. She decides on a model, but she does not know at which specific prices it is available.^2^ Based on other purchases of subjectively similar products, she surmises that 600 euros would be an acceptable price, and decides to get at least three quotes. She visits a store, at which the refrigerator is offered for 800 euros; feeling that her initial reservation price may have been unrealistic, she becomes willing to pay 700 euros. She visits the manufacturer’s website and finds a price of 1000 euros, revising her reservation price to 750 euros. In an online shop, she finds a price of 720 euros. Her reservation price (for this and as an anchor for similar products) falls to 730 euros. Given the apparent variability of prices, she identifies one more quote of again 800 euros. Her reservation price remains at 730 euros, and she goes back to the online shop to order the refrigerator for 720 euros, 20 percent more than her initial reservation price (Figure 2).

## 4. Method

### 4.1. Experimental Procedure

To collect data, I conducted an online laboratory experiment in which subjects went through simulated shopping situations. By following a personalized link, subjects accessed the experimental website from their own devices.^3^ Instructions and displays are shown in Appendix A.

The task in each situation was to purchase an experimental good from one of a number of sellers. The nature of the good was unspecified to prevent confounding effects arising from home-grown preferences or perceived product quality (see Scitovszky, 1944; Smith, 1976); the goods were identified solely with randomly preselected letters. For instance, one situation asked subjects to buy a good labeled “Product G” (labels and descriptions are translated from German).

At the beginning of each situation, subjects were informed about their initial monetary endowment ($B_{0}$), the resale value of the good ($\tilde{P}$), and the cost of visiting a seller (*C*). Subjects moved on to an “overview page.” This page listed placeholders for a number ($\tilde{T}$) of sellers, which were labeled “Seller 1,” “Seller 2,”…, “Seller $\tilde{T}$.” To visit a seller’s shop to identify its price, subjects had to click on a placeholder. The sequence in which prices were shown was randomly preselected to control exposure to the experimental treatments (see also Kogut, 1992; Schotter & Braunstein, 1981; Schunk, 2009; Sonnemans, 1998). To increase external validity by avoiding forced choices, the overview page allowed subjects to cancel the situation without making a purchase.

All shops had an identical parsimonious design and consisted of a single page showing the seller’s name and the price charged ($P_{t}$). The names were randomly preselected letters, e.g., “Seller E.” Although using highly simplified shops may reduce external validity, it helps in eliminating extraneous variables such as perceived website quality. The shops contained two buttons labeled “Buy from this seller” and “Do not buy from this seller.”

When subjects visited a shop without making a purchase, they were taken back to the overview page. The placeholder was replaced with the seller’s name and its price; e.g., “Seller 2” was replaced with “Seller E offers the product for a price of EUR 100.00.” Subjects could select another placeholder to visit the next shop or return to any previous shop, i.e., exercise a recall. When subjects made a purchase or canceled a situation, they entered the next situation.

### 4.2. Incentive Structure

Subjects entered a lottery of 15 shopping vouchers for 20 euros. The probability of winning depended on performance in the experiment through Equation (8).

(8) $$B_{T}=B_{0}-C\times T-P+\tilde{P}$$

$B_{T}$ is a subject’s final balance in a situation; $B_{0}$ is the initial endowment; *C* is marginal search cost; *T* is the total number of sellers visited; *P* is the price paid for the good; and $\tilde{P}$ is the induced value of the good. All variables except *T* are in euros as the experimental currency.

Specifically, subjects started each situation with 600 euros ($B_{0}$). For every seller visited (*T*), 5 or 20 euros were subtracted (*C*); recalls were free. Given the imposition of search costs, subjects had an incentive not to visit too many sellers. When a subject made a purchase, the price was subtracted (*P*). Hence, subjects had an incentive to search for a low price and not to stop their search too early. Upon making a purchase, 100 or 400 euros were added ($\tilde{P}$).

After the study was concluded, one situation was selected at random. The probability of winning a voucher was proportional to a subject’s $B_{T}$ in that situation.

### 4.3. Operationalization and Measurement of the Reservation Price

To be more specific than up to this point, a consumer’s reservation price at time *t*, $RP_{t}$, is the maximum amount of money the consumer is willing to pay for a good at that time. The terms “reservation price” and “willingness to pay” can be considered synonyms, with more common use of the first term in economics (e.g., Varian, 1992) and of the second term in marketing research (e.g., Wertenbroch & Skiera, 2002).

Since $RP_{t}$ cannot be observed directly, I used the common procedure by Becker et al. (1964) to measure it. This “BDM-mechanism” is incentive-compatible and has been shown to have high validity and reliability (e.g., Wertenbroch & Skiera, 2002). The mechanism consists of three steps. First, the subject states their current willingness to pay for the good ($RP_{t}$). Second, a price $P_{\mathrm{BDM}}$ is drawn at random. Third, the subject automatically buys the good if $RP_{t}\geq P_{\mathrm{BDM}}$ and does not buy it if $RP_{t}<P_{\mathrm{BDM}}$. When a “BDM-purchase” occurs, the subject is charged $P_{\mathrm{BDM}}$ (not $RP_{t}$).

The BDM-mechanism was administered repeatedly in each situation to collect successive measurements of $RP_{t}$(see also Cox & Oaxaca, 1992; Schotter & Braunstein, 1981): Before a subject visited the first seller (t=0), the initial $RP_{t}$ was measured. After a subject had identified a price and not made a purchase (t=1,2,…,T−1), $RP_{t}$ was measured again. After a purchase (t=T), $RP_{t}$ was not measured again since the present research does not cover post-purchase behavior.

### 4.4. Experimental Design and Experimental Rounds

The experimental design is defined by the manipulation of $P_{t}$ (sellers’ prices) as the main independent variable and manipulations of ancillary independent variables $\tilde{P}$, *C*, and $\tilde{T}$ (induced value, search cost, number of sellers). The ancillary manipulations were included because a pretest suggested the possibility of insufficient variation in the dependent variable $RP_{t}$ and, hence, the possibility of a false negative due to a restriction of range (Nunnally & Bernstein, 1994, p. 130; Cohen et al., 2003, p. 57). Moreover, covering a range of situations increases generalizability.

The ancillary manipulations follow a 2×2×2 factorial design, which results in eight different shopping situations, as shown in Table 1. Given the heterogeneity of real shopping situations, there is no objective basis to select the values of $\tilde{P}$, *C*, and $\tilde{T}$; thus, they were chosen to represent varied yet reasonable settings (see also Kogut, 1992; Telser, 1973).

For the manipulation of $P_{t}$, values were randomly preselected: for each situation, $\tilde{T}$ values were drawn from a normal distribution with $\mu =0.9\times \tilde{P}$ and σ=0.15×μ. The random values were rounded to multiples of five to yield prices not ending in psychologically relevant or obscure figures. The resulting prices are shown in Table 2.

The experiment uses a within-subjects design, i.e., each subject was exposed to all experimental rounds. This design allowed an increase in statistical efficiency by modeling unobserved heterogeneity. To control for order effects, the sequence in which subjects were exposed to the rounds was rotated with a sequentially balanced Latin square design.

### 4.5. Supplementary Rounds

In addition to the eight situations described above, four trivially altered replicas of these situations, counterbalanced between subjects, were included for methodological purposes (see also Schotter & Braunstein, 1981): One replica was placed at the end of the experiment for a manipulation check and to assess the goodness of the measurement of the reservation price. Three replicas were added at the beginning and during the experiment to strengthen the credibility of the BDM-mechanism. In the eight experimental rounds but not in the replicas, BDM-purchases were precluded to avoid truncating the sample, as otherwise, subjects with a relatively high $RP_{t}$ would finish a round more often within the BDM-mechanism than subjects with a relatively low $RP_{t}$. In addition, these three replicas also serve to assess the goodness of the measurement of the reservation price. The results of the methodological checks are reported in Appendix B.

To practice using the experimental interface, subjects could complete up to five trial runs, one being mandatory. Trial conditions were different from those in the experimental rounds to prevent learning effects.

### 4.6. Sample Characteristics

A market research company was commissioned to recruit 450 participants. The sample size was based on cost considerations. The company drew gender–age stratified random samples from its panel of consumers until the sample size was reached. Subjects who completed the experiment received 3 euros. A total of 22 subjects generated contaminated data (i.e., the data set contained several observations on $RP_{t}$ for the same search step or a $B_{T}$ that was inconsistent with behavior), resulting in an effective sample of 428 subjects. The mean time to complete the experiment was 19.7 min, with a standard deviation of 8.8. A total of 45 percent of the subjects were female, and 55 percent male. Age ranged from 15 to 79 years, with a mean of 37 and a standard deviation of 14.

Given the participants’ membership in a market research panel, the sample was not a random sample from the population. Nevertheless, the sample should be sufficiently broad for the results to apply to a relatively wide range of consumers; it does not rely on university members.

## 5. Results

### 5.1. Descriptive Statistics and Treatment of Outliers

Table 3 summarizes behavior and performance in the experimental rounds. Subjects went through 3424 shopping situations, with 3344 ending in a purchase. A total of 98.5 percent of the purchases were made at the best price observed; 9.7 percent involved a recall. On average, subjects visited 2.34 sellers (not counting recalls), paid a price of 226.57 euros, and had a final balance of 594.58 euros.^4^ The latter value shows that on average, subjects would have achieved a higher balance (of $B_{0}=600$) by avoiding search costs, i.e., not visiting sellers. This was unknown to the subjects ex ante, as it is in real shopping situations.

Table 4 summarizes the observations on $RP_{t}$ that were generated with the BDM-mechanism. There are 8396 observations, ranging from 0 to 1000 euros. It is possible that very low and very high values were “the result of gross deviation from prescribed experimental procedure” (Grubbs, 1969, p. 1; see also Camerer & Hogarth, 1999; Cohen et al., 2003, p. 411f.). Therefore, I conducted an outlier analysis, explained in Appendix C. The analysis identified 440 severe outliers. As imputing the “correct” value would be based on speculation, these observations were discarded (see Grubbs, 1969; Cohen et al., 2003, p. 415). For the remaining 7956 observations, the mean is 214.26, the median is 250.00, and the standard deviation is 133.53. The main conclusion from this estimation (Section 5.3) is robust to the inclusion of outliers.

### 5.2. Regression Models

#### 5.2.1. Main Specification

The main model to be tested is Equation (7) (Section 3.2). A corresponding econometric specification is a Regression Model (RM) (RM1):

(RM1) $$\begin{array}{cc} RP_{ijt}=\beta_{0} & +\beta_{1}RP_{ij,t-1}\\ & +\beta_{2}^{+}\left[\max\left(0,P_{jt}^{\min}-RP_{ij,t-1}\right)\right]\\ & +\beta_{2}^{-}\left[\min\left(0,P_{jt}^{\min}-RP_{ij,t-1}\right)\right]\\ & +\nu_{i}+\epsilon_{ijt} \end{array}$$

The dependent and independent variables of theoretical interest are defined as in Section 3.2; *i* indexes subjects, *j* simulated shopping situations, and *t* search steps. To control for unobserved heterogeneity, $\nu_{i}$ is included as a fixed effect (e.g., Wooldridge, 2010, pp. 285–309). Since $\nu_{i}$ is not of interest in itself, I apply a within transformation to (RM1) (and the other models specified below). The transformation allows for $\beta_{0}$ to be interpreted in the usual way as the model constant. The within-transformed (RM1) is estimated using ordinary least squares.

#### 5.2.2. Alternative Specifications

To evaluate (RM1) against alternatives, I specify four additional regression models. (RM2) is an econometric specification of Equation (6) as follows:

(RM2) $$RP_{ijt}=\beta_{0}+\beta_{1}RP_{ij,t-1}+\beta_{2}\left(P_{j,t-1}^{\min}-RP_{ij,t-1}\right)+\nu_{i}+\epsilon_{ijt}$$

(RM3) is (RM1) without separating positive and negative adjustments of the reservation price, as follows:

(RM3) $$RP_{ijt}=\beta_{0}+\beta_{1}RP_{ij,t-1}+\beta_{2}\left(P_{jt}^{\min}-RP_{ij,t-1}\right)+\nu_{i}+\epsilon_{ijt}$$

While these two models primarily serve to assess the refinements from Section 3.2, they can also be justified on different theoretical grounds. (RM2) can be reexpressed such that it portrays the reservation price as a weighted moving average, similar to the model by Levinthal and March (1981; see Lant, 1992), or representing a reference-dependent updating process in which the reservation price adjusts towards past performance. (RM3) is structurally equivalent to a dynamic anchoring-and-adjustment mechanism, a Koyck (1954) lag specification, or an adaptive expectations model, depending on the properties of the error term.

(RM4) models a constant reservation price, as predicted by neoclassical search theory when consumers know the price distribution (Section 2.1):

(RM4) $$RP_{ijt}=\beta_{0}+\beta_{1}RP_{ij,t-1}+\nu_{i}+\epsilon_{ijt}$$

When consumers do not know the distribution, the reservation price depends positively on the mean ($\bar{P}$) and negatively on the standard deviation ($SD^{p}$) of the known prices (see Kohn & Shavell, 1974; Rothschild, 1974). In addition, the reservation price rises in marginal search cost (*C*) and in the number of search steps (*t*), the latter because search only continues when high prices are observed and beliefs about the distribution become more pessimistic (Casner, 2021; Dubra, 2004; Kohn & Shavell, 1974; McCall, 1970; Rothschild, 1974; Telser, 1973; Weitzman, 1979). (RM5) models this setting as follows:

(RM5) $$RP_{ijt}=\beta_{0}+\beta_{1}{\bar{P}}_{jt}+\beta_{2}SD_{jt}^{p}+\beta_{3}C_{j}+\beta_{4}t_{ij}+\nu_{i}+\epsilon_{ijt}$$

Although these two models do not allow for a conclusive comparison of the optimizing vs. satisficing theories of search, the academic reception of neoclassical models warrants at least a basic assessment.

### 5.3. Estimation Results

Table 5 shows five model selection criteria: the information criterion by Akaike (1974; AIC), the Bayesian information criterion by Schwarz (1978; BIC), the root mean squared error (RMSE), within R^2^, and adjusted within R^2^. Since (RM2) includes $P_{j,t-1}^{\min}$ and (RM5) includes $SD_{jt}^{p}$ (which requires at least two prices), the estimation samples cannot encompass the first observation per search spell. Therefore, Table 5 is split between these models and the other models, which can be estimated using all available data. The table also includes the results for (RM1) using the smaller sample, denoted as (RM1)’.^5^ The criteria show that the main specification consistently outperforms the alternative specifications.

The estimation results for (RM1) are shown in Table 6. The results for the other models are relegated to Appendix D.

Since the parameter hypotheses from Section 3.2 are directional, one-sided *p*-values are appropriate (while Table 6 shows two-sided):

- $b_{0}<0$: The estimate $\beta_{0}=-2.584$ is significantly smaller than zero (t(411)=−3.55, p=0.0002).
- $0<b_{1}\leq 1$: $\beta_{1}=0.977$ is significantly greater than zero (t(411)=257.19, p<0.0001) and smaller than one (t(411)=−6.10, p<0.0001).
- $0<b_{2}^{+}\leq 1$ and $0<b_{2}^{-}\leq 1$: $\beta_{2}^{+}=0.353$ is significantly greater than zero (t(411)=13.12, p<0.0001) and smaller than one (t(411)=−24.08, p<0.0001). For $\beta_{2}^{-}=0.781$, results are mixed: the estimate is significantly greater than zero (t(411)=2.46, p=0.0073) but not smaller than one (t(411)=−0.69, p=0.2457). The latter result is driven by cluster-robust standard errors; with conventional standard errors, the estimate is significantly smaller than one (t(4316)=−3.78, p<0.0001). Thus, the lower-bound part of the hypothesis is supported, while the upper-bound part is supported under the assumption of independent and identically distributed error terms. The lower-bound part is sufficient for downward adjustments of the reservation price.
- $b_{2}^{+}<b_{2}^{-}$: A similar qualification applies for $\beta_{2}^{+}=0.353<\beta_{2}^{-}=0.781$. With cluster-robust standard errors, the difference is weakly significant (t(411)=1.30, p=0.0969), while it is significant with conventional standard errors (t(4316)=7.13, p<0.0001). The difference seems large enough to deem it practically important.

## 6. Discussion

### 6.1. Summary

The results suggest that the short-term dynamics of consumers’ reservation prices during the search for prices under uncertainty can be explained behaviorally on the basis of the ADM by Lewin et al. (1944) and, more generally, the theory of aspiration levels in the spirit of its application to economic phenomena by Simon (1955): Consumers adjust their reservation prices to the prices identified during search. Specifically, the reservation price in a search step is based on the previous reservation price and the difference between the lowest price identified so far and the previous reservation price. Equation (9) summarizes the results with the estimates in Equation (7), the main model of the present research, as follows:

(9) $$\begin{array}{cc} RP_{t}=-2.58 & +0.98RP_{t-1}\\ & +0.35\left[\max\left(0,P_{t}^{\min}-RP_{t-1}\right)\right]\\ & +0.78\left[\min\left(0,P_{t}^{\min}-RP_{t-1}\right)\right] \end{array}$$

**Figure 3:**
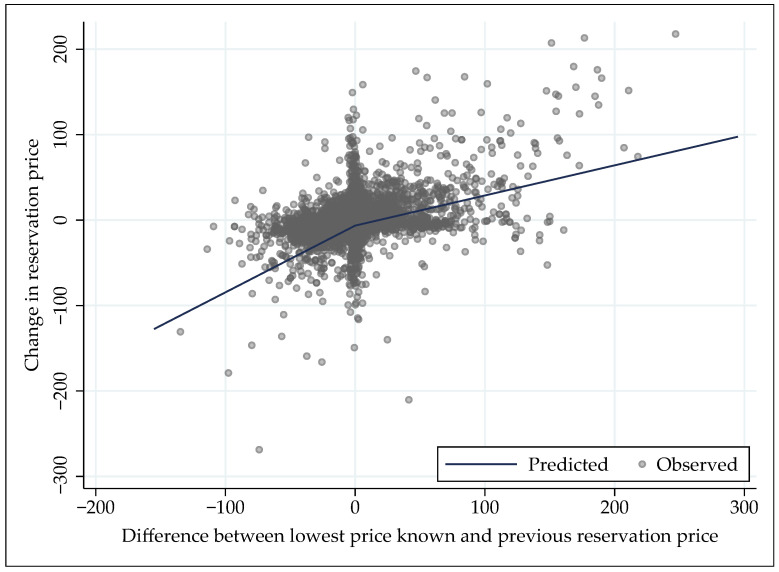
Adjustment of the reservation price to the lowest price known. Note: Added-variable plot with predicted and observed values conditioned on other independent variables in econometric specification (RM1).

Ceteris paribus and on average, subjects reduced their reservation price in the experiment by 2.58 euros from one search step to the next. The tendency to reduce reservation price also manifests itself in less-than-complete inertia, i.e., for each 1 euro increase in the previous reservation price, the reservation price in a search step increases by 98 cents. The core of this model shows that there is an asymmetric adjustment to the prices identified during search: when the lowest price known was higher than the previous reservation price, each 1 euro increase in the positive difference pushed the reservation price up by 35 cents; when the lowest price known was lower than the previous reservation price, each 1 euro increase in the negative difference pulled the reservation price down by 78 cents (Figure 3).

### 6.2. Implications

#### 6.2.1. Behavioral Challenges to Welfare Economics

When the reservation price adapts to the prices identified during search, it not only reflects the marginal utility the consumer associates with the good but also the terms at which it can be bought.^6^ The difference between reservation prices and prices paid is therefore not consumer surplus in the conventional sense. Instead, in mental accounting terms (Thaler, 1985, 1999), the difference combines acquisition utility, which is consumer surplus, and transaction utility, which reflects the attractiveness of the terms of an exchange relative to potential other exchanges of the good. This does not invalidate microeconomic reasoning. If consumers care not only about “value for money” (acquisition utility) but also about “a good deal” (transaction utility), then both confer benefit, and institutions that increase either enhance welfare.

Yet, the interpretation of reservation prices shifts. Consumers may often know little about how much a good is “worth” (to them), even when they are not unsure of its quality. Their assessment of a good’s value depends on how much is charged for it by sellers, which stands in contrast to the neoclassical notion that a good’s value is how much consumers are willing to pay for it. Preferences depend on prices, and prices depend on preferences and the consumer behavior they give rise to. This circular causality and the resulting dynamic endogeneity have been noted in the literature (e.g., Ariely et al., 2006; Gilboa & Schmeidler, 2001; Isoni et al., 2016; Mazar et al., 2014), and the present results reinforce this view.

Such behavioral findings, i.e., consumers being influenced by supposedly irrelevant factors, are regularly interpreted to justify government intervention and skepticism towards maximizing consumer sovereignty (e.g., Ariely et al., 2003, 2006), notwithstanding cautioning remarks (e.g., Thaler, 2015) and discussions (e.g., Cowen & Dold, 2021; Epstein & Rizzo, 2018; Rizzo & Whitman, 2019). An indispensable step in evaluating such arguments is to determine which supposedly irrelevant factors are in fact relevant, and this step should be guided by theory to avoid “ad hocery” (Conlisk, 1996, p. 685). Reservation prices may be influenced by, for example, social security numbers in experiments (Section 2.2), but such anchors are not salient in market transactions; prices are, as suggested by the theory of aspiration levels (and other approaches from Section 2; see also, for example, Ariely et al., 2006; Mazar et al., 2014). The question then is under which conditions prices as salient anchors result in reservation prices that maintain the beneficialty of consumer sovereignty. The unexacting answer is that it depends on the market’s structure.

Specifically, the optimal price for a seller is lower when consumers have reservation prices (or reference prices; Winer, 1988), but it is higher when reservation prices adjust upward to higher prices. Thus, the present results may be construed as an argument for paternalistic policies to protect consumers against the sellers’ incentive to profit from upward adaptations of the reservation price, but such protection can be offered by competition: competitive markets increase the likelihood that consumers observe low prices during search, which results in downward adaptations and therefore keeps reservation prices, and thus optimal prices for sellers, in check. Conversely, lack of competition and collusive practices allow sellers to benefit from upward adaptations. More generally, “changes in the level of aspiration…tend to keep goals consistent with reality” (Starbuck, 1963, p. 56), and for consumers, reality is shaped by the degree of competition between sellers. Hence, the conviction that competition results in allocatively efficient prices is another microeconomic reasoning that remains intact.

#### 6.2.2. Theoretical Foundations of Behavioral Economics

Behavioral economics has benefited from uncovering biases in decision making, i.e., systematic departures from expected utility theory (e.g., Tversky & Kahneman, 1974). Augmenting models of optimal behavior by incorporating such biases (e.g., Kogut, 1990, explores the sunk cost effect, and Dubra, 2004, overconfidence in sequential search) may enhance their ability to explain observed and predict future behavior. Nonetheless, while this option to account for biases may be considered to be in line with bounded rationality, the starting point is still perfect rationality (e.g., Altman, 2023; Simon, 1979). A different option is to base models on alternative theories that provide deeper and more structured explanations. Using theories that do not assume optimal behavior as a starting point may introduce the potential for ad hocery as they may be lacking in mathematical rigor, but this potential is lower compared to “bias patching” of optimizing models, especially since for many biases, there may be a corresponding opposite bias (e.g., the sunk cost effect may lead to escalation of commitment or the opposite—both over- and underconfidence are possible; see also Gigerenzer, 2018; Simon, 1984). The present research constitutes an attempt to use an alternative theory as the starting point to model consumer search behavior under bounded rationality (see also Gigerenzer & Selten, 2001; Selten, 1998).

A prominent behavioral alternative to expected utility theory is prospect theory (Kahneman & Tversky, 1979; Tversky & Kahneman, 1992). The theory of aspiration levels includes several elements of prospect theory, albeit in a less accessible and rigorous manner (see Lewin et al., 1944).^7^ Moreover, the ratio between the coefficients for negative and positive adjustments of the reservation price in Equation (9) is 0.78/0.35=2.23. This estimate of “failure aversion” in the theory of aspiration levels (Section 3.2) is almost identical to an estimate of loss aversion in the prospect theory of 2.25 (Tversky & Kahneman, 1992, see also Tversky & Kahneman, 1991; Thaler, 1999). Since asymmetric behavioral phenomena are observed in different contexts (e.g., Ahrens et al., 2017; Isoni et al., 2016; Mezias, 1988; Murphy et al., 2001; Schunk, 2009; Tversky & Kahneman, 1991), theoretical explanations are valuable, and in this respect, the theory of aspiration levels is compatible with prospect theory. In addition, the former sheds some light on a question on which the latter is largely silent (e.g., Puto, 1987; Winer, 1988): what determines the reference point in decision making, and how does it change over time? In prospect theory, the reference point is typically taken to be the status quo or the adaptation level, but it can also be related to an aspiration level (Kahneman & Tversky, 1979, 1984; Antonides, 1996; Gilboa & Schmeidler, 2001; Mezias, 1988; Murphy et al., 2001; Puto, 1987; Thaler, 1999; Tversky & Kahneman, 1991). Since the theory of aspiration levels aims at explaining the performance an individual aspires to attain, it adds insight by treating the aspiration level not only as an independent but also as a dependent variable (Section 3.1).

### 6.3. Limitations

Since these findings are based on an online laboratory experiment, it can be questioned whether they can be generalized to a natural environment. A field study is needed to establish external validity. For example, while there was a financial incentive in the experiment (see Section 4.2), the subjects’ own money was not at stake, which may have an unpredictable effect on the adjustment of the reservation price.

Additional questions remain. Which settings lead searchers to *not* purchase a good even if, in principle, acceptable prices are observed? In the experiment, subjects who canceled a situation had a strong tendency to reduce their reservation price when they identified a lower price—to the point of “overshooting cheapskate behavior” in that a difference of one euro made them want to find a price more than two euros lower. This implies that reservation prices can become negative and, generally, that aspiration levels can rise too much for success to be possible (see Lewin et al., 1944). Investigating this issue would be informative, as it may prevent Pareto-optimal transactions. What determines the initial reservation price (which was treated as exogenous here) in a search spell? In addition to marginal search cost, past experiences may be particularly important if consumers generalize from one spell to another for a related good. Such carry-over or “transfer” effects are an element of the theory of aspiration levels (Lewin et al., 1944, p. 339), and show conflicting evidence (e.g., Brown et al., 2011; Casner, 2021; Hey, 1982; Hoeffler & Ariely, 1999; Isoni et al., 2016; Nunes & Boatwright, 2004). Carry-over effects imply that during their economically formative years and over time, consumers acquire a set of reservation prices that serve as a basis for subsequent purchase situations. This would imply that reservation price adjustments have long-term effects, while here, short-term adjustments were studied. Which factors determine the limit to upward adjustments of the reservation price? The consumer’s budget constraint offers only a partial answer, as the allocation of funds to future consumption may not reflect an intertemporal optimization—saving may be a residual category, receiving funds only after current consumption desires are fulfilled. A good’s “true” or “intrinsic” value also does not offer a complete answer, as there may not be an actual construct in consumer behavior that is equivalent to the induced value from experimental methodology. When reservation prices are influenced by the prices charged in the market, it is may be questioned whether consumers have a latent and yet definitive monetary valuation of the good.

More generally, the model and results presented here show *how* the reservation price changes during search, but not *why*, as they do not uncover the psychological mechanism driving the adaptation. The behavioral dynamics may stem from a conscious, deliberate type of processing or an unconscious, automatic effect, or a mixture of both. Similarly, while the experiment relied on unspecified goods to control for perceived product quality, natural markets may trigger a price–quality heuristic: the reservation price may fall (or, analogously, rise) because consumers associate lower prices with lower quality and devalue the product category. Because the ADM can accommodate these varied latent processes, disentangling them is a possible venue for future research.

## 7. Conclusions

The reservation price is a central construct in the microeconomic theory of demand, as it determines which goods consumers are willing to buy (e.g., Varian, 1992). In neoclassical microeconomics, the reservation price reflects the marginal utility of a good to the consumer and, hence, a stable preference ordering. Neoclassical search theory enriches this conceptualization by allowing consumers to update their reservation price by rationally responding to the information environment (e.g., Rothschild, 1974). This, however, can only be based on the as-if assumption, since the computations required are daunting and cannot be performed by real-life consumers (see also Hey, 1981).

An alternative is the satisficing heuristic (Simon, 1955, 1956), the “poster child” of bounded rationality (Artinger et al., 2022, p. 598). Under satisficing, decision makers choose an alternative that is “the best so far” or “good enough,”, i.e., an alternative that meets the current level of aspiration with respect to the alternatives’ attributes. This level of aspiration serves the same function as the reservation price, as both separate acceptable from unacceptable alternatives. However, the level of aspiration is not presumed to be the solution of a mathematical problem, but rather a psychological construct.

When Simon introduced satisficing, he referred to this foundation in the psychological theory of aspiration levels, but only commented briefly on its “dynamic considerations” (Simon, 1955, p. 111). In the present article, such considerations are explored in the context of sequential price search. The main model of this research is based on the attainment discrepancy model by Lewin et al. (1944) as a semi-formalization of the theory of aspiration levels. The results from an online laboratory experiment with more than 400 participants from the general population lend support to the model.

The results suggest that reservations prices may not reflect stable preferences or a rational response to the information environment. Instead, the model postulates a simple adjustment process: the reservation price shifts towards the prices encountered during search. The adjustment is asymmetric in that upward adaptations are less strong than downward adaptations: consumers are likely to become willing to pay more when they encounter high prices, and even more likely to become willing to pay less when they encounter low prices. Specifically, in the experiment, when the lowest price known was higher than the previous reservation price, the reservation price rose by 35 cents per euro of the difference; for prices below the reservation price, it fell by 78 cents per euro. This adjustment does not require consumers to solve a mathematical problem, and its simple nature seems to concur with casual observation.

These findings can contribute to our understanding of consumer decision making in dynamic choice situations under uncertainty. From a theoretical perspective, this article indicates the value of alternative frameworks as a starting point rather than patching optimization models with catalogued biases. From a policy perspective, the results have implications for consumer welfare in markets, as they suggest that when sellers charge higher prices, consumers become willing to pay more. These behavioral dynamics imply opportunities for sellers to extract consumer surplus. Yet, they do not imply an argument for paternalistic economic policies, as competition between sellers can protect consumers: by exposing consumers to low prices during search, competitive markets shift reservation prices downwards, limiting the sellers’ ability to exploit adaptiveness. In turn, lack of competition results in such an ability. Thus, the findings provide a behaviorally grounded rationale for competition policy that complements standard arguments.

Open questions remain. First, a field study is needed to establish external validity. Second, future research could investigate conditions under which reservation prices overshoot to fall beyond attainability, the determinants of the initial reservation price in a search spell, carry-over effects across search spells, the limits to adjustments of the reservation price, and the psychological mechanisms underlying the adaptation.

In summary, the reservation price can be conceptualized behaviorally as a level of aspiration that adjusts towards the lowest price known: consumers satisfice in that they attempt to find a price that is “good enough,” but what is “good enough” depends on the prices charged in the market. It is not dismal to conclude that competition can offer sufficient protection for consumers against sellers exploiting malleable reservation prices.

## Figures and Tables

**Table 1:** Conditions in the shopping situations used as experimental rounds.

		$\tilde{P}$			
		100		400	
		C		C	
		5	20	5	20
$\tilde{T}$	5	S1	S2	S3	S4
	10	S5	S6	S7	S8

Note: $\tilde{P}$: induced value of the good in euros; *C*: marginal search cost in euros; $\tilde{T}$: number of sellers; S*x*: simulated shopping situation *x*.

**Table 2:** Prices in the experimental rounds.

	$P_{t}$									
t	1	2	3	4	5	6	7	8	9	10
S1	100	90	75	110	75					
S2	90	110	75	95	100					
S3	380	420	325	355	315					
S4	415	380	365	375	400					
S5	90	90	105	75	95	70	75	90	85	95
S6	100	110	90	85	85	115	80	95	90	90
S7	370	430	365	405	345	385	370	400	405	340
S8	415	370	385	355	400	335	320	330	365	405

Note: $P_{t}$: price charged by seller *t* in euros; Sx: simulated shopping situation *x*.

**Table 3:** Descriptive statistics on subject behavior during the experimental rounds.

	No. of Purchases	No. of Purchases at $P_{T}^{\min}$	No. of Purchases by Recall	Mean No. of Visited Sellers ($\bar{T}$)	Mean Purchase Price ($\bar{P}$)	Mean Final Balance ($\bar{B_{T}}$)
S1	421	419	11	2.29	85.69	602.61
S2	420	411	45	1.78	85.25	578.91
S3	425	419	48	2.35	347.28	640.60
S4	415	411	48	2.42	377.61	573.29
S5	417	413	42	2.39	84.41	603.24
S6	409	403	47	2.40	93.64	557.99
S7	421	408	49	2.64	364.76	621.44
S8	416	408	35	2.47	371.17	578.59
Total	3344	3292	325	2.34	226.57	594.58

Note: $P_{T}^{\min}:=\min\left(P_{1},P_{2},\ldots ,P_{T}\right),\bar{P}$, and $\bar{B_{T}}$ in euros; Sx: simulated shopping situation x;n=428 subjects.

**Table 4:** Descriptive statistics on subjects’ reservation prices in the experimental rounds.

	No. of Observations	Mean	Median	Standard Deviation	Minimum	Maximum
S1	996	85.18	78.50	60.25	2.00	1000.00
S2	810	92.84	80.00	69.06	1.00	750.00
S3	1054	320.21	345.00	76.98	1.00	1000.00
S4	1094	329.06	350.00	74.60	0.00	800.00
S5	1071	84.05	75.00	60.14	0.00	800.00
S6	1086	92.06	80.00	63.24	1.00	600.00
S7	1185	319.78	330.00	69.68	1.00	600.00
S8	1100	330.88	350.00	69.91	1.00	600.00
Total	8396	213.25	250.00	136.51	0.00	1000.00

Note: Values except no. of observations in euros; Sx: simulated shopping situation x;n=428 subjects.

**Table 5:** Model selection criteria for main and alternative specifications.

Model	AIC	BIC	RMSE	$R^{2}$	$R_{adj}^{2}$
n=4731					
(RM1)	40,597.66	40,617.05	17.66	0.9800	0.9800
(RM3)	40,651.08	40,664.00	17.76	0.9797	0.9797
(RM4)	42,548.18	42,554.64	21.71	0.9697	0.9697
n=2544					
(RM1)’	20,760.99	20,778.51	14.31	0.9852	0.9851
(RM2)	21,049.57	21,061.25	15.15	0.9834	0.9833
(RM5)	22,647.92	22,671.29	20.73	0.9689	0.9688

Note: (RM1) and (RM1)’: main specifications; (RM2)–(RM5): alternative specifications.

**Table 6:** Estimation results for the main specification.

Dependent Variable: $RP_{ijt}$				
Independent Variable	Coefficient	Estimate	Standard Error	*p*-Value
	$\beta_{0}$	−2.584	0.728	0.0004
$RP_{ij,t-1}$	$\beta_{1}$	0.977	0.004	0.0000
$\max\left(0,P_{jt}^{\min}-RP_{ij,t-1}\right)$	$\beta_{2}^{+}$	0.353	0.027	0.0000
$\min\left(0,P_{jt}^{\min}-RP_{ij,t-1}\right)$	$\beta_{2}^{-}$	0.781	0.318	0.0145
Observations (subjects)	4731 (412)			
*F*-statistic	72,182.26			
Degrees of freedom	3, 411			
Model *p*-value	0.0000			

Note: Subject-level fixed effect included via within transformation, including cluster-robust standard errors.

**Figure A1:**
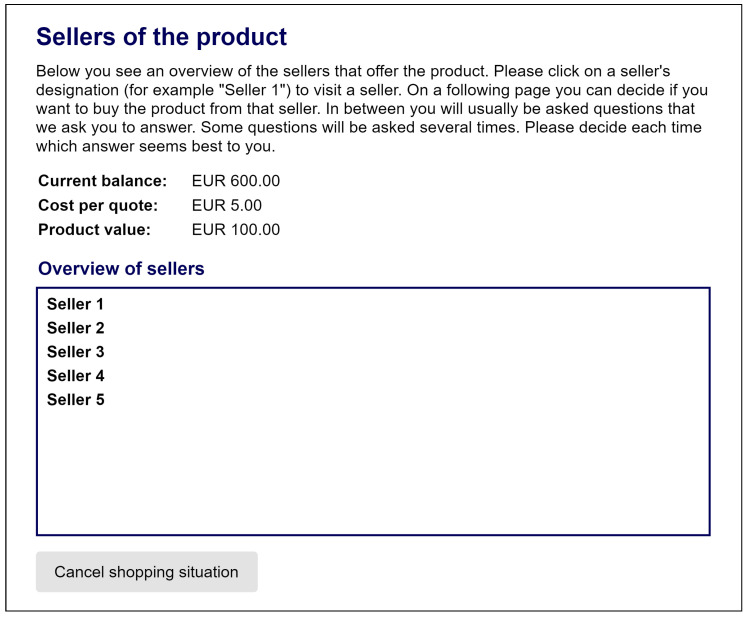
An illustration of the “overview page” at the beginning of a situation.

**Figure A2:**
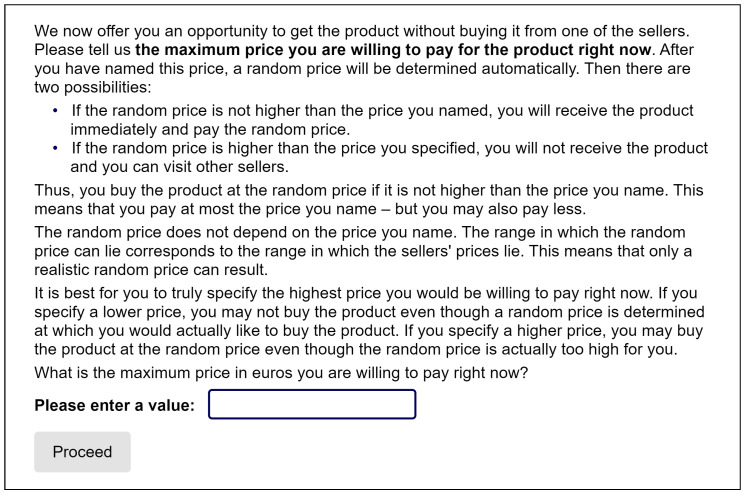
An illustration of the BDM-mechanism.

**Figure A3:**
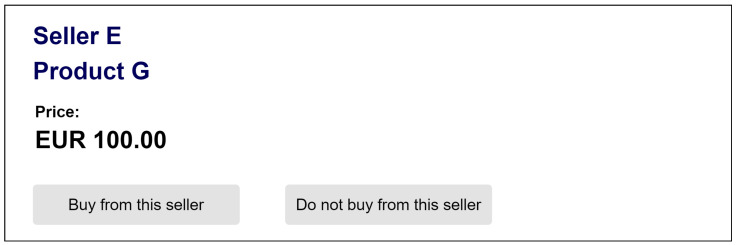
An illustration of a seller’s shop.

**Figure A4:**
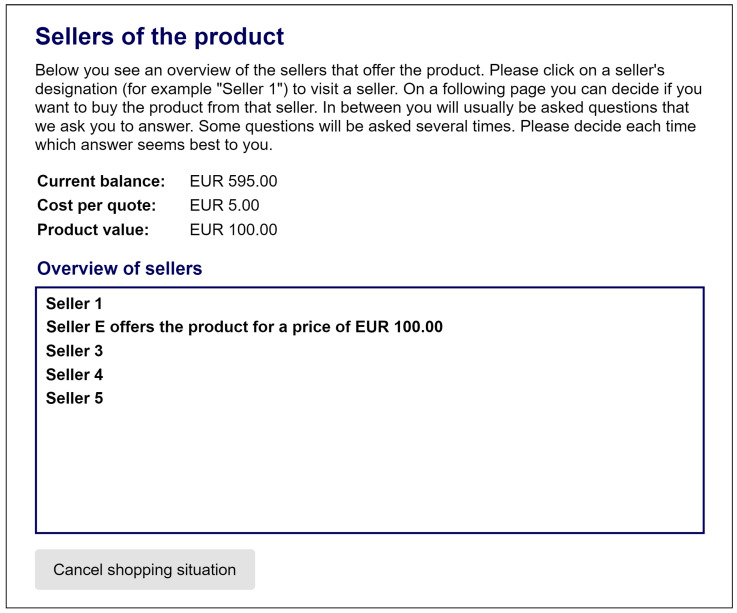
An illustration of the “overview page” during a situation.

## Data Availability

The dataset is available online at http://doi.org/10.17632/hzfcg7z832.1.

## Footnotes

1 Generically, aspiration levels have an upward orientation: high values are preferred to low values. In the terminology by Lewin et al. (1944, p. 335), an individual’s “ideal goal” is the maximum that is possible. In consumer price search, the ideal goal is the minimum, and low values are preferred to high values. See also Selten (1998).

2 It would be more general to assume that the consumer, when deciding on a specific product, also incorporates information about prices. For the present research, I assume the simpler setting, i.e., analytically separate the decision on quality from the decision on price, and model only the latter.

3 Since existing software could not be used, especially because of the need to repeatedly measure the reservation price, the website was developed for this research and related projects. Prior results were published in, for example, van Baal (2011, 2015). A part of the present explanations is based on these works.

4 The descriptives align with previous findings. Similar proportions of subjects not making a purchase were reported by Hey (1982) and Kogut (1992). The number of sellers visited is consistent with the recurring observation that many consumers tend to search little (Casner, 2021; Johnson et al., 2004; Kogut, 1990; Marcu & Noussair, 2024; Schunk, 2009; Schunk & Winter, 2009; Sonnemans, 1998; Zwick et al., 2003)

5 In total, 7956 reservation prices for 428 subjects are available (Section 5.1). In (RM1), (RM3), as well as (RM4), the lagged reservation price reduces the estimation sample by 3077 initial values, and by another 148 because of a lagged outlier, resulting in 4731 observations for 412 subjects. For (RM1)’, (RM2), and (RM5), another 2187 first observations per search spell cannot enter the estimation, resulting in 2544 observations for 372 subjects.

6 A similar implication follows from neoclassical search models with an unknown price distribution (Section 2.1), as they allow for changes in beliefs about the distribution to affect reservation prices.

7 As a case in point, compare the diagram in Siegel (1957, p. 257) to the value function in Kahneman and Tversky (1979, p. 279).

## Appendix A. Experimental Instructions and Displays

The following texts and illustrations are translated from German.
*Before registration:* Thank you for your interest in our study on decision-making behavior. By taking part, you are making an important contribution to scientific research and at the same time you can win an online-shopping voucher for 20 euros! The study is very simple: Your task is to buy a specific product. To do this, you can visit a number of simulated sellers and then decide where to buy the product.
You will not make actual buying commitments in this study. Nevertheless, it is important that you behave in the same way as you would if you were making a real purchase. The simulated sellers are based on sellers that also exist in reality: In the study, real-life conditions are replicated as closely as possible.
To take part in the study, all you need to do is register. We need some personal data to make statistical statements. Your e-mail address will only be used to inform you if you have won a shopping voucher. On the next page, you will find an explanation of how the study works. Thank you in advance for your support!
*After registration:* In this study, you will encounter several simulated shopping situations. In each situation, your task is to buy a specific product. It is not important which product it is. The products are therefore referred to as “Product A” or “Product D,” for example. Just imagine that you have decided to buy a certain product that can be used over a longer period of time.
The product is offered by several sellers in each shopping situation. You can visit these sellers and then decide where to buy the product. Just like the products, the sellers are not mentioned by name, but are referred to as “Seller A” or “Seller D,” for example. The sellers and the product do not differ within a shopping situation! All sellers therefore offer the same product. However, the sellers may charge different prices.
Some information is important for you in every shopping situation:
  - The starting balance: This is the euro amount you have at the start of the situation.
  - The cost per quote: For each seller you visit, this euro amount will be deducted from your balance. However, you can visit a seller repeatedly without being charged again.
  - The price paid: When you buy the product, the price charged by the chosen seller will be deducted from your balance.
  - The product value: This is the euro amount that will be added to your balance when you buy the product. Imagine that you resell the product at the product value immediately after you have bought it.
  - The final balance: This is the euro amount you have left at the end of a situation.
An example: You have a starting balance of 1000 euros in a shopping situation, the cost per quote is 10 euros, and the product value is 500 euros. You visit five sellers and buy the product from one of them for 400 euros. This leaves you with 1050 euros as your final balance, according to the following calculation:
  	1000 euros	(starting balance)
  −	5 × 10 euros	(number of sellers visited times cost per quote)
  −	400 euros	(price paid)
  +	500 euros	(product value)
  =	1050 euros	(final balance)
All participants have a chance to win one of 15 online-shopping vouchers for 20 euros. You can influence your chance of winning by earning a high final balance in each shopping situation: After the study is concluded, we will randomly select one situation—and for every euro of your final balance in that situation, one ticket will enter the prize pot for you!
Your task in every situation is to buy the product. However, you can also cancel a situation without buying the product. In this case, no price paid will be deducted from your balance, but the product value will not be credited to you either.
You can take as much time as you need to complete the study, but you should not take a break of more than 10 min. The average completion time without a break is approximately 20 min. You will be informed when you have completed all the shopping situations.
If you have any questions, please contact: *researcher contact information*.

## Appendix B. Validity, Reliability, and Manipulation Checks

### Appendix B.1. Measurement Validity

Given that the reservation price $RP_{t}$ is the study’s focal construct, it is sensible to assess the goodness of its measurement with the BDM-mechanism (Section 4.3) as adapted for the experiment. For instance, the mechanism was administered repeatedly, as in Becker et al. (1964), but unlike many other studies. Two tests of predictive criterion validity are based on behavior in the experimental rounds, and a supplementary round is used for a test of convergent construct validity (see Nunnally & Bernstein, 1994, pp. 83–113).

The first test of predictive criterion validity is the correlation between the price paid (criterion) and the measured $RP_{t}$ before the purchase (predictor). Spearman’s rank correlation is 0.855 and is significantly different from zero (t(3130)=92.27, p<0.0001). A lower acceptance limit of 0.3 or 0.4 is common (Nunnally & Bernstein, 1994, p. 99f.). The second test is the hit rate for the prediction that when a subject does not make a purchase in a search step (criterion), $P_{t}^{\min}$ should be higher than $RP_{t}$ (predictor) in most cases (see also Schotter & Braunstein, 1981). The reliance on non-purchases and the qualification “in most cases” reflect Stopping Condition 1 (Section 3.3): some subjects continue to search even after identifying an acceptable price. The hit rate is 92.9 percent. While this is significantly lower than 100 (binomial test, p<0.0001), Stopping Condition 1 explains the non-congruent cases. To perform a simple test of convergent construct validity, I elicited subjects’ price acceptability judgments (e.g., Lichtenstein et al., 1988) at the beginning of the last supplementary round and correlated it with the subsequent $RP_{t}$. This judgment was measured as the agreement to the statement “$0.9\times \tilde{P}$ euros would be an acceptable price for the product” ($0.9\times \tilde{P}$ was 90 or 360, depending on the situation) on a scale from “1: Disagree completely” to “7: Agree completely.” Spearman’s rank correlation is 0.489. While the correlation is modest, possibly because the judgment was measured with a single item, its direction is as expected and it is significantly different from zero (t(374)=10.85, p<0.0001). These three tests support the hypothesis that the BDM-mechanism, as used in the experiment, provides a valid measurement of $RP_{t}$.

### Appendix B.2. Measurement Reliability

To assess the reliability of the measurement of $RP_{t}$, the data from the eight experimental rounds is correlated with the data from the four supplementary rounds. This procedure yields an estimate of test–retest reliability, as the supplementary rounds were counterbalanced replicas of the experimental rounds: subjects provided data on $RP_{t}$ in S1 through S8 and also in an almost identical subset. Spearman’s rank correlation for the matched measurements is 0.849 and is significantly different from zero (t(2511)=80.55, p<0.0001). A lower acceptance limit of 0.7 or 0.8 is common (Nunnally & Bernstein, 1994, p. 264f.). While the methodological literature tends to disfavor test–retest reliability as a way to evaluate measurement consistency, the criticism that subjects might remember their earlier responses and wish to *appear* consistent is unlikely to apply here because subjects would need to remember a large number of prices and recognize the similarity of situations (see Nunnally & Bernstein, 1994, p. 254f.).

### Appendix B.3. Manipulation Check

The main independent variable in the experiment is the sellers’ prices $P_{t}$. Although $P_{t}$ is observable, it is prudent to check whether the manipulation was successful in the sense that subjects paid attention to the prices. To perform a simple manipulation check, I measured subjects’ perceived price variability (e.g., Dolansky & Vandenbosch, 2013) after the second price was shown in the last supplementary round with the single item “The prices I have seen thus far in this shopping situation differ” on a scale from “1: Disagree completely” to “7: Agree completely.” In the replica of S5, the first two prices are identical (Table 2), and the median of the subjects’ responses is 1. In the replicas of the other situations, the first two prices are not identical, and the median is 6. The responses are significantly different (Wilcoxon rank-sum test, z=−7.26, p<0.0001). Furthermore, Spearman’s rank correlation for the range of the two prices and perceived price variability is 0.408 and is significantly different from zero (t(182)=6.03, p<0.0001). This check provides convergent evidence of a successful manipulation.

## Appendix C. Detection of Outliers

The outlier analysis for the data on $RP_{t}$ consisted of two steps: First, I used the rule by Tukey (1977, p. 43f.) to identify severe outliers, i.e., values that deviate more than three times the interquartile range downwards from the first quartile (lower threshold) or upwards from the third quartile (upper threshold). Second, since Tukey (1977)’s rule is exploratory, I used the test by Walsh (1959; see also Hawkins, 1980, p. 83f.) to inferentially determine whether the values identified in the first step belong to the same population as the other values. This procedure has the advantages of being nonparametric, not relying on an assumed model of the data-generating process, and identifying only highly suspect values as outliers.

These steps were performed separately for situations with $\tilde{P}=100$ euros (S1, S2, S5, S6) and $\tilde{P}=400$ euros (S3, S4, S7, S8), since $RP_{t}$ is significantly different between these two groups (Wilcoxon rank-sum test, z=−72.77, p<0.0001). Nonparametric tests are used since the null hypothesis that $RP_{t}$ stems from a normal distribution is rejected across all situations (skewness–kurtosis test by D’Agostino et al., 1990, $\chi^{2}\left(2\right)=19,871.41$, p<0.0001) and within the two groups of situations ($\chi^{2}\left(2\right)=4295.83$, p<0.0001 for $\tilde{P}=100$ euros and $\chi^{2}\left(2\right)=1689.07$, p<0.0001 for $\tilde{P}=400$ euros). For $\tilde{P}=100$ euros, the interquartile range is 15 euros; the lower threshold is 25 euros and identifies 39 severe outliers; and the upper threshold is 130 euros and identifies 220 severe outliers. For $\tilde{P}=400$ euros, the interquartile range is 60 euros; the lower threshold is 120 euros and identifies 167 severe outliers; and the upper threshold is 540 euros and identifies 14 severe outliers. For all four sets of outliers, the test by Walsh (1959) rejects the null hypothesis that they are from the same population as the other values (p<0.05). Thus, in total, 440 observations on $RP_{t}$ were identified as severe outliers.

## Appendix D. Additional Estimation Results

**Table A1:** Estimation results for specifications other than (RM1).

Coefficient	Estimate Standard Error (*p*-Value)				
	(RM1)’	(RM2)	(RM3)	(RM4)	(RM5)
*β*_0_	−2.542	−1.850	−2.958	4.822	−13.403
	0.812	0.661	0.666	0.524	1.761
	(0.0019)	(0.0054)	(0.0000)	(0.0000)	(0.0000)
*β*_1_	0.989	0.981	0.973	1.009	0.913
	0.004	0.005	0.003	0.002	0.010
	(0.0000)	(0.0000)	(0.0000)	(0.0000)	(0.0000)
*β*_2_		0.256	0.371		−0.620
		0.030	0.025		0.099
		(0.0000)	(0.0000)		(0.0000)
$\beta_{2}^{+}$	0.296				
	0.035				
	(0.0000)				
$\beta_{2}^{-}$	0.802				
	0.185				
	(0.0000)				
*β*_3_					−0.050
					0.075
					(0.5036)
*β*_4_					1.553
					0.503
					(0.0022)
Observations	2544	2544	4731	4731	2544
Model *p*-value	0.0000	0.0000	0.0000	0.0000	0.0000

Note: Subject-level fixed effect is included via within transformation, including cluster-robust standard errors..
